# Monogenic Diabetes with GATA6 Mutations: Characterization of a Novel Family and a Comprehensive Analysis of the GATA6 Clinical and Genetics Traits

**DOI:** 10.1007/s12033-023-00761-8

**Published:** 2023-05-18

**Authors:** Xing Yue, Yaheng Luo, Jing Wang, Debin Huang

**Affiliations:** Department of Metabolism and Endocrinology, The Third Hospital of Changsha, Laodongxi Road #176, Changsha, 410011 Hunan People’s Republic of China

**Keywords:** Diabetes, GATA6, De novo mutation, Pancreas, Genotype–phenotype correlations

## Abstract

Monogenic diabetes caused by GATA6 mutations were almost described as neonatal diabetes, and the phenotypic spectrum has expanded since then. Our study underscores the broad phenotypic spectrum by reporting a de novo GATA6 mutation in a family. Furthermore, we reviewed related literature to summarize the clinical and genetic characteristics of monogenic diabetes with GATA6 mutations (*n *= 39) in order to improve clinicians’ understanding of the disease. We conclude that the GATA6 missense mutation (c. 749G > T, p. Gly250Val) is not reported presently, characterized by adult-onset diabetes with pancreatic dysplasia and located in transcriptional activation region. Carries with GATA6 mutations (*n *= 55) have a variable spectrum of diabetes, ranging from neonatal (72.7%), childhood-onset (20%) to adults-onset (7.5%). 83.5% of patients with abnormal pancreatic development. Heart and hepatobillary defects are the most common abnormalities of extrapancreatic features. Most mutations with GATA6 are loss of function (LOF, 71.8%) and located in functional region. Functional studies mostly support loss-of-function as the pathophysiological mechanism. In conclusion, there are various types of diabetes with GATA6 mutations, which can also occur in adult diabetes. Phenotypic defects with GATA6 mutations are most frequently malformations of pancreas and heart. This highlights the importance of comprehensive clinical evaluation of identified carriers to evaluate their full phenotypic spectrum.

**Supplementary Information:**

The online version contains supplementary material available at 10.1007/s12033-023-00761-8.

## Introduction

Monogenic diabetes, which accounts for 1–5% of diabetes cases [1], including neonatal diabetes, maturity-onset diabetes of the young and monogenic diabetes syndromes [2]. So far, more than 30 subtypes have been identified in these monogenic diabetes with specific clinical phenotypes and genetic patterns. Most of them coding proteins which play critical roles in pancreatic and beta cell development and function [3, 4]. Monogenic diabetes is frequently misdiagnosed and treated as either type 1 or type 2 diabetes [5]. Thus, getting a correct diagnosis for monogenic diabetes has important implications for management, prognosis and counseling of an individual’s diabetes and other family members regarding likely inheritance.

GATA6-binding factor 6 (GATA6), a zinc finger transcription factor, is crucial in pancreatic development. It is important in the differentiation of endoderm, the development of pancreatic fate, the generation of endocrine/exocrine cells and the function of mature endocrine/exocrine cells [6, 7]. In 2011, a large number of GATA6 gene mutations were detected in the cohort of pancreatic underdevelopment, which was the first clinical study to find that GATA6 plays an important role in pancreatic development [8]. As a rare type of monogenic diabetes, GATA6 mutations is mostly reported as cases. In the past, it was believed that the GATA6 mutation was mainly manifested as neonatal diabetes, which is an important mutation gene for neonatal diabetes. With the development of gene diagnosis technology, in recent years, a few reports have found that monogenic diabetes with GATA6 mutations can be manifested in children and even adults [8, 9], but there is no report of adult diabetes as the proband. In addition to pancreatic development abnormalities, GATA6 mutations can also show different pancreatic abnormalities, including congenital heart defects and some abnormalities originating from the endoderm, such as hepatobiliary deformities, gallbladder dysplasia and intestinal hernias [8–10]. It can be seen that the clinical characteristics of the GATA6 mutation are significantly heterogeneous. At the same time, even family members with the same GATA6 allele mutation have different clinical manifestations [11–13], showing significant phenotype-clinical differences, which brings great challenges to the diagnosis of GATA6 mutant monogenic diabetes.

We report a de novo GATA6 mutation (c. 749G > T, p. Gly250Val) family with paternal inheritance. The proband is characterized by adult-onset diabetes, pancreatic agenesis. His daughter just presents with pancreatic abnormality. This illustrate the phenotypic heterogeneity associated with GATA6 mutations. Moreover, we firstly outline 39 different GATA6 mutations with pancreatic dysfunction and/or developmental defect have been reported in the literature so far and explore the genotype–phenotype correlations of them, in order to improve clinicians’ understanding of the disease.

## Methods and Materials

### Clinical Evaluation and Genetic Testing

The proband with adult-onset diabetes and pancreatic agenesis was recruited at our hospital. Whole-exome sequencing was performed in the proband, his wife and daughter on DNA isolated from peripheral blood leucocytes at the Beijing Genomics Institute (Shenzhen, China). Detailed phenotypic data were obtained by medical history interviews and from clinical records.

## Literature Search

We searched English papers in PubMed using “GATA6 Transcription Factor” [Mesh] AND “GATA6 protein, human” [Supporting Information Concept] until March 2021. This resulted in 225 items. Suitable papers were selected based on the abstract. Inclusion criteria were: GATA6 mutations patients with diabetes and/or pancreatic developmental defect where the full text was available. Exclusion criteria were: no evidence of diabetes, no genetic information, no full text. We checked the references in these papers and screened papers that cited the included papers. Lastly, we scanned Clinvar and OMIM to identify any missing mutations. We summarized and analyzed the genotype–phenotype correlations of GATA6 mutations patients which described in 21 papers [8–28] (in the supplement). Statistical comparisons were performed using Fisher's exact test, and Bonferroni corrected p values were used for determination of statistical significance (*p *= 0.01).

## Results

### Clinical Descriptions of the Proband and His Daughter in the De Novo GATA6 Mutation Family

**Fig. 1:**
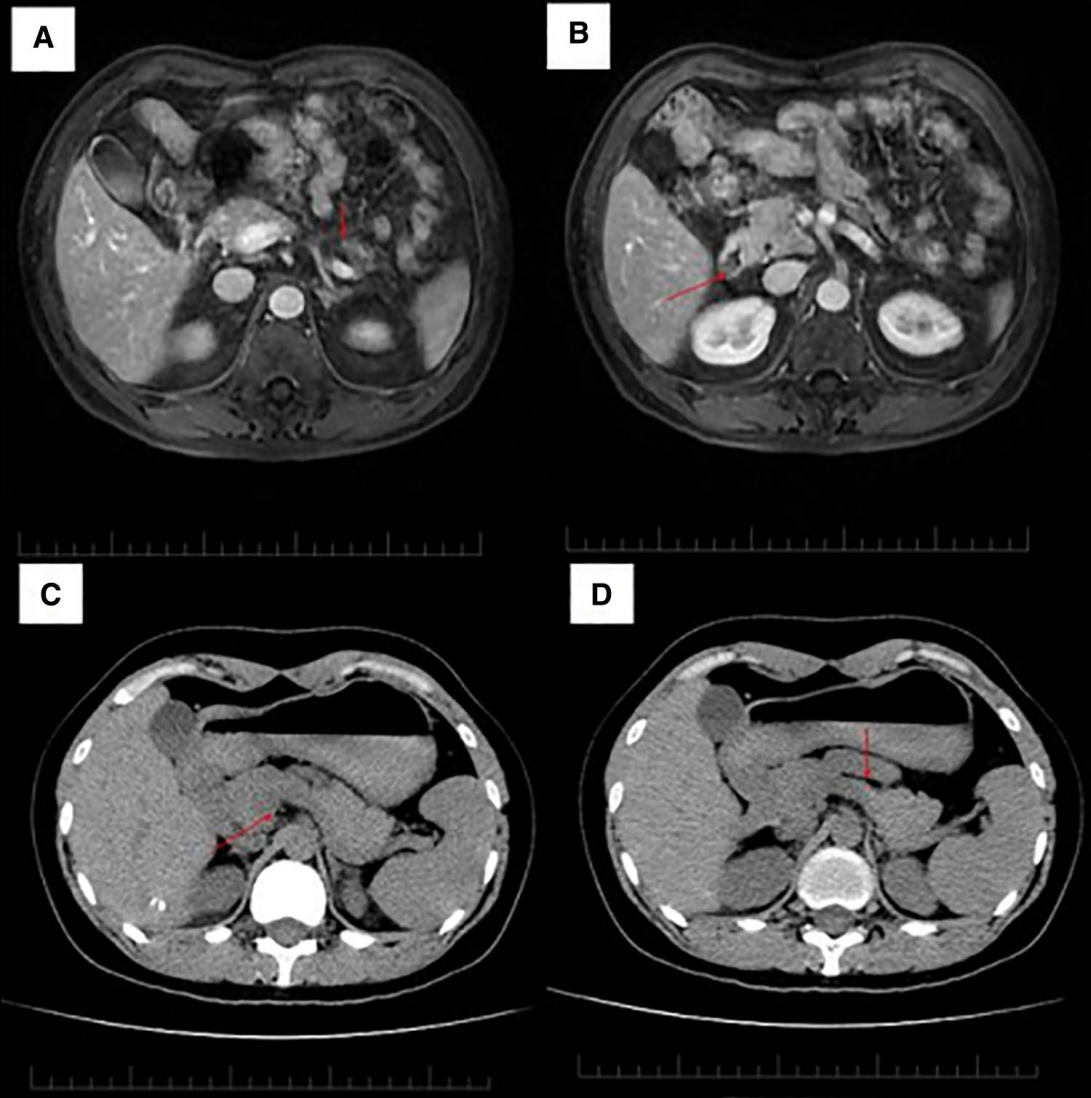
Imaging of pancreas in the proband and his daughter. Magnetic resonance imaging of the proband showing only the head of the pancreas is visible (**A, B**). Computed tomography scan of his daughter with pancreatic body hypoplasia (**C, D**). Abnormalities are indicated by red arrows

The proband, a 62-year-old male born to healthy nonconsanguineous parents, was diagnosed with type 2 diabetes mellitus (DM) for 4 years. He visited our department for hypoglycemia with abnormalities in insulin secretion. Discovery of the GATA6 mutation initiated a renewed examination of the etiology of the DM and revealed pancreatic dysplasia (Fig. 1A, B). In addition, his daughter presented with pancreatic abnormality and normal glucose tolerance (NGT) at her oral glucose tolerance test (OGTT) (Fig. 1C, D). Clinical and laboratory findings and follow-up characteristics of them are described in Table 1. He was able to obtain good glycemic control with acarbose.

**Table 1:** Overview of clinical phenotypes of the patient and his daughter

	The proband	His daughter
Current age	62	26
Sex	Male	Female
History of DM	4 years	No
HbA1c (%)	6	5.1
Fasting glucose (mmol/l)	6.71	4.67
Postprandial glucose (mmol/l)	15.53	6.78
Fasting insulin (3.0–25.0 uU/ml)	1.50	5.96
Postprandial insulin (uU/ml)	60.3	61.86
GAD-Ab, IAA-Ab, ICA-Ab	Negative	Negative
Pancreatic phenotype	Hypoplasia	Abnormality
Echocardiography	Normal	Normal
Other defects	No evidence	No evidence
Pancreatic exocrine function	No evidence	No assessment
Treatment of DM	Changing gliclazide with acarbose to Acarbose	No

Clinical description of basic information, diabetes, pancreatic, cardiac, and other phenotypes in the patient and his daughter. The proband with adult-onset diabetes treated by acarbose and pancreatic agenesis. His daughter just presents pancreatic abnormality

### Genetic Testings of the De Novo GATA6 Mutation Family

**Fig. 2:**
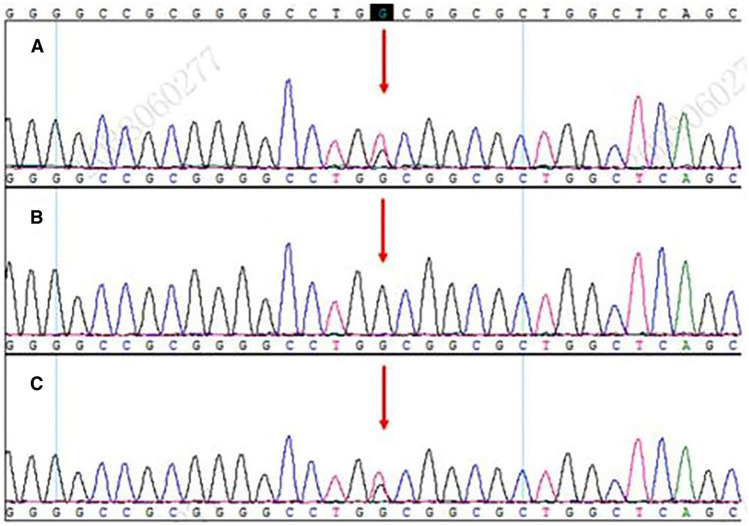
Genetic testings of the de novo GATA6 mutation family. **A** The proband. **B** His wife. **C** His daughter. The proband and his daughter with GATA6 heterozygous mutation (NM_005257.4;c. 749G > T;p. Gly250Val)

The results of whole-exome sequencing in the proband, his wife and daughter (Fig. 2). The same GATA6 mutation (c. 749G > T, p. Gly250Val, chr18:19751854) was identified in the proband and his daughter. At present, such mutation is either classified as variants of unknown significance (VUS) or is not reported.

### The Genotype–Phenotype Analysis of GATA6 Mutations with Pancreatic Dysfunction and/or Developmental Defect in the Literature

#### Clinical Traits

There were 39 GATA6 mutations, including nonsense, frameshift, splice site and misense mutations, in 45 independent proband. In seven probands, the mode of inheritance was not tested. In the remaining, the variant arose de novo in 73.6% (28/38). Inherited mutations were found in 10 cases, of which maternally expressed [8, 11, 12, 15, 27] and paternally expressed [8, 16] each account for 50%. So, GATA6 can be a de novo mutation or family inheritance, most are the former. No difference between maternal and paternal expression.

#### Phenotypic Manifestations

**Fig. 3:**
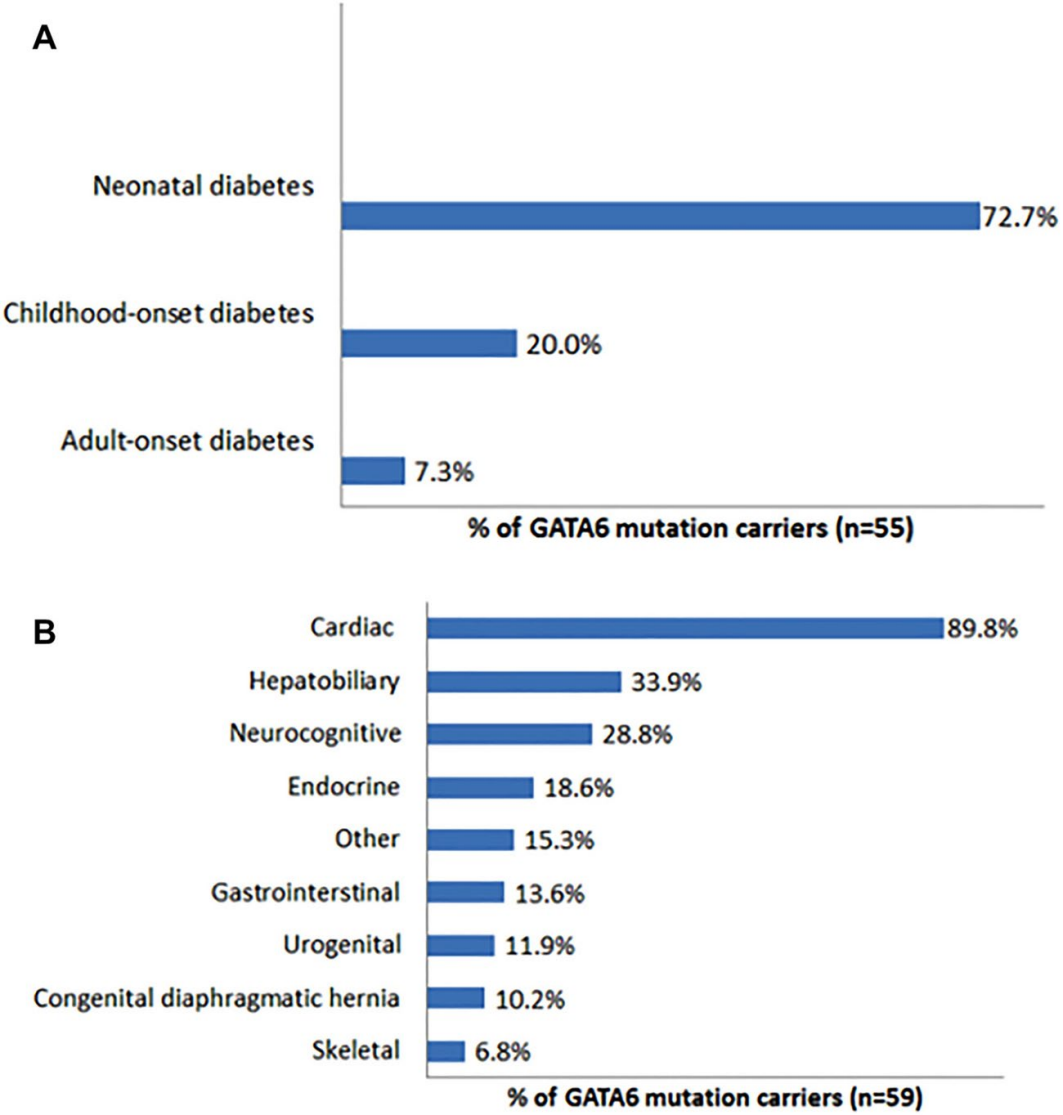
Clinical phenotypes of GATA6 mutations carriers. **A** The incidence of monogenic diabetes in 55 carriers. **B** The incidence of extrapancreatic abnormalities in 59 carriers. Carries with GATA6 mutations (*n *= 55) have a variable spectrum of diabetes, ranging from neonatal (72.7%), childhood-onset (20%) to adults-onset (7.5%). Heart (89.8%) and hepatobillary (33.9%) defects are the most common abnormalities of extrapancreatic features

In total, there were data available on 59 GATA6 mutation carriers (45 probands and 14 family members from 10 families) with available pancreatic dysfunction and/or developmental defect in 55 of them. 96.3% of the carriers (*n *= 55, Fig. 3A) exhibited diabetes mellitus. Among them, 72.7% manifested neonatal diabetes mellitus, 20.0% manifested childhood-onset diabetes mellitus and 7.3% manifested adults-onset diabetes mellitus. Two cases with transient neonatal diabetes developed permanent diabetes mellitus in 3 and 5 years old [12, 24]. There were 83.6% with agenesis of the pancreas and 67.3% with exocrine pancreatic insufficiency in carriers (*n *= 55). The most common other extrapancreatic features were heart (89.8%, mainly ventricular septal defect) and hepatobiliary (mainly gallbladder agenesis) in 59 GATA6 mutation carriers. Diaphragmatic hernia with an incidence of 10.2% and hypothyroidism was the most frequently reported endocrine dysfunction in these cases. Therefore, the clinical manifestations of GATA6 are diverse and significantly heterogeneous.

#### Genotype–Phenotype Correlations

We stratified the patients according to inheritance (de novo vs. inherited) and found that patients with a de novo mutation more often presented neonatal diabetes mellitus and pancreas agenesis than those with an inherited mutation (Table S2, *p *< 0.01). In mutation families, all probands had neonatal diabetes mellitus and pancreas agenesis, clinical and phenotypic diversity resulted from family members. Members displayed different clinical phenotypes in the same mutation family, thus, the phenotype and genotype do not correlate. Intriguingly, carriers without pancreatic dysfunction and/or developmental defect occurred within maternal inheritance families (4/5), three of them were parents [11, 12, 15, 27] and the remaining was younger brother [12]. Four were adult-onset diabetes [8, 16] and one was childhood-onset diabetes [8] mellitus in paternal inherited families. No significant differences were found between maternally and paternally inheritance in other abnormalities. Inter-sex differences also were not detected.

**Fig. 4:**
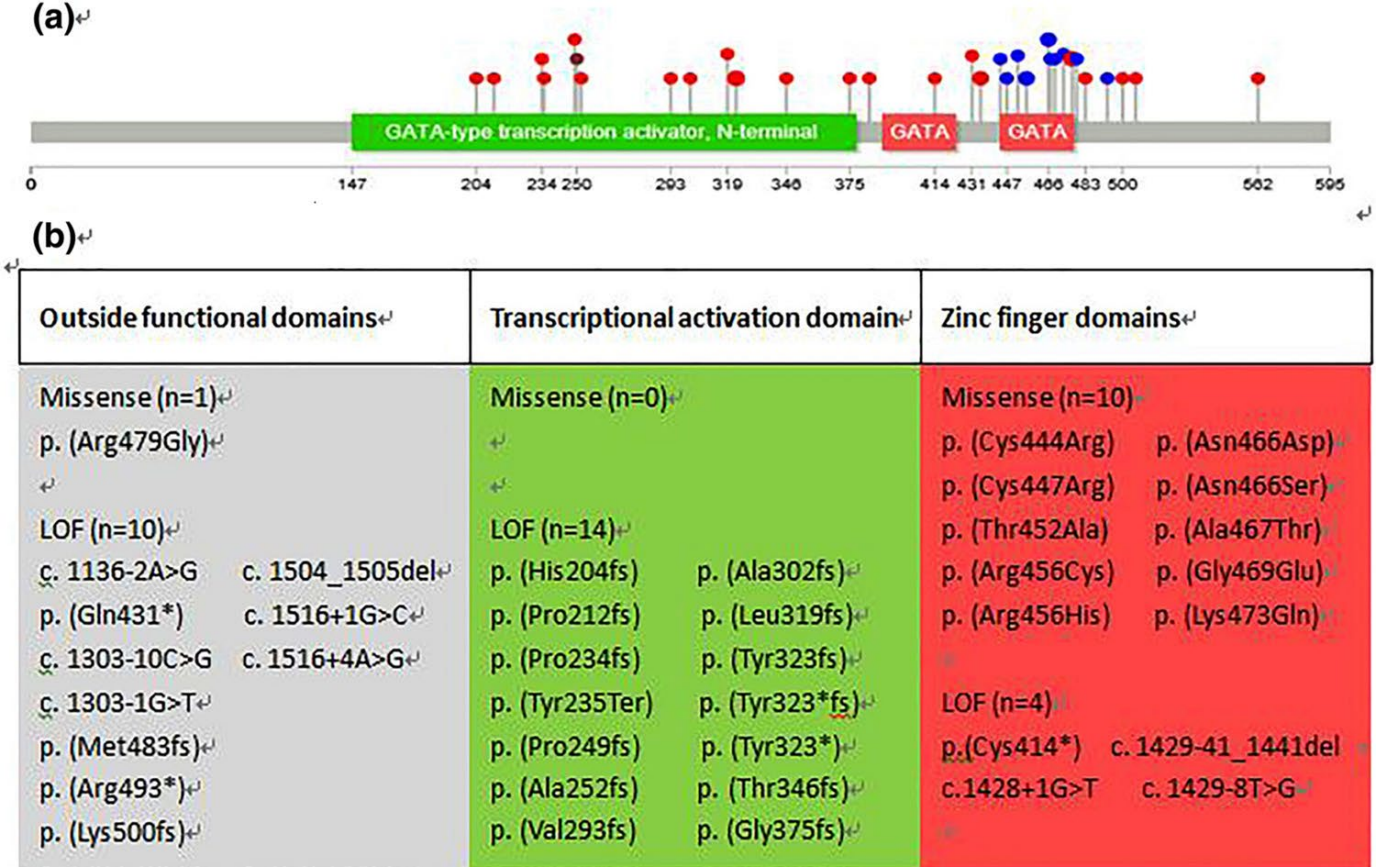
Presentations of the GATA6 protein and its protein domains with all described mutations. **a** A diagram of the GATA6 protein with the functional domains “N-terminal” (GATA-type transcription activator) and “GATA” (zinc finger domains). Our case and 39 mutations have been reported are plotted at their topological location on the GATA6 protein. Blue dots represent missense mutations, Red dots represent loss-of-function mutations, and brown dot represent our mutation. Splice site mutations are plotted at the closest amino acid position. Bigger dots represent different mutations at the same amino acid position. The figure is created using The Lollipops software [39], which uses the Pfam protein domain database (http://pfam.xfam.org/) to retrieve domains. **b** Overview of all 39 mutations categorized by position on the protein. Most mutations were located within the functional domains of GATA6 (28/39, 71.8%), most of them are LOF (28/39, 71.8%)

When stratifying patients according to the type of mutation (misense vs. LoF mutation, Fig. 4), we did not observe any statistically significant differences in the terms of pancreatic dysfunction and/or developmental defect (Table S3).

Four mutations were found in more than one independent proband (c. 1242C > A [14, 18], c. 1366C > T [9, 19, 26], c. 1367G > A [9, 20], c. 1504_1505del [11, 13]) and one of these, c. 1366C > T p. (Arg456Cys) was even found in four independent probands. It is located in the C-zinc finger domain of the protein, essential for modifying the DNA-bind function by active with GATA6 protein. All of these four probands varied in other extrapancreatic defects with pancreas agenesis and cardiac abnormalities. Three of them presented neonatal diabetes, the remaining was childhood-onset diabetes. Two probands had pancreatic exoendocine dysfunction.

Therefore, the genotype and phenotype of the GATA6 mutation are heterogeneous, and there is no relationship between the two.

#### Genic Location of GATA6 Mutations

Topologically, the GATA6 protein has at least three functional domains: a transcriptional activation domain essential for its transcriptional activity and two zinc finger domains (C- and N-) required for DNA sequence recognition and binding to the consensus motif [29]. We plotted all mutations on a diagram of the GATA6 protein (Fig. 4). Most mutations were located within the functional domains of GATA6 (28/39, 71.8%). Furthermore, misense mutations were more often located in the zinc finger domains, whereas LOF mutations were more often located in the transcriptional activation domain. There were no statistically significant differences in clinical manifestations between mutations located inside and outside of the functional domains. The mutation we have reported was a miseen, located in transcription activation domain, as far no reported. Functional studies mostly support loss-of-function as the pathophysiological mechanism.

## Discussion

More and more studies have found that GATA6 plays an important role in the formation, differentiation, maturation, and function of the endoderm and pancreas. GATA6 encodes a highly conserved zinc finger transcription factor, identifies and combines (A/T) GATA (A/G) to regulate the base sequence, forms a complex with other transcription factors to regulate chromatin structure [30, 31], increases transcriptional activity, and upregulates mRNA expression, in the endoderm and pancreatic It plays an important role in cell formation. GATA6 mutations cause changes in retinol dose, abnormal Hedgelog signaling pathways, and accumulation of H3kemel to reduce DNA methylation inhibition [32–34], resulting in abnormal convoids and a decrease in the number of pancreatic progenitor cells. Studies [26, 35] show that GATA6 mutations cause apoptosis/proliferation abnormalities of islet cells, abnormal structure of B cell endoplasmic reticulum and mitochondria, and the increase of immature insulin particles, which affect the production and secretion of insulin, and abnormal endocrine function, showing different types of diabetes. GATA6 mutation leads to follicular apoptosis/proliferation abnormalities, follicular catheterization, and fat cell transdifferentiation, resulting in exocrine functional defects that occur independently of endocrine dysfunction [36]. Therefore, the formation, differentiation, maturation, and dysfunction of the endoderm and pancreas caused by GATA6 mutations is an important pathological basis for GATA6 mutation monogenic gene diabetes.

Monogenic diabetes is caused by splicing site, meaninglessness, de novo meaning or transcoding mutation. More rarely, it is partial or complete deletion, which affects a single gene. The phenotype and related extrapancreatic characteristics vary according to the affected gene [37]. Our genotype and phenotypic analysis of GATA6 mutant monogenic diabetes shows that there is significant heterogeneity in both clinical phenotype and genotype–phenotype correlation. This heterogeneity may be due to differences in genetic background, environmental factors and epigenetic factors, which is related to haploid insufficiency, chimeric, incomplete octropy, and mutation time window [8, 9, 12, 30, 38]. With the development of genetic technology, the genotypes and phenotypes of monogenic diabetes are becoming more and more abundant, and the significant heterogeneity of monogenic diabetes poses a challenge to the early clinical identification of such diseases. Therefore, we have summarized the clinical and genetic characteristics of GATA6 mutations, hoping that clinicians can solve the relevant information and make corresponding clinical decisions.

In the past, it was known that monogenic diabetes with GATA6 mutation often manifested as neonatal diabetes and abnormal pancreatic development. More and more reports have found that monogenic diabetes with GATA6 mutation can manifest as diabetes in children or adults, with or without pancreatic developmental defects, which can be combined with heart defects, hepatobiliary defects Abnormalities such as muscle hernia, growth retardation, hypothyroidism, etc. Neonatal diabetes caused by GATA6 mutation is insulin dependence, but it is sensitive to insulin. A small dose of insulin (0.15–0.5u/h) is enough, and glucose fluctuates easily. A small number of adolescents or adults used hypoglycemic drugs (mainly metformin) in the early stage of diabetes, and then gradually changed to insulin [8, 18, 21]^.^ In this case, the new GATA6 mutant paternal family shows adult diabetes and pancreatic dysplasia. At present, oral acarbose blood sugar control is good. The daughter only shows pancreatic dysplasia, and only pancreatic involvement cases have been reported in the past [8]. At present, there is no evidence of pancreatic exocrine glands and pancreatic defects. In one case of GATA6 mutation, protein lossy bowel disease occurred in the course of the disease [8, 20]. Therefore, whether the patient and daughter will have the progress of the disease still needs to be closely observed. In addition, a progressive defect of glucose homeostasis was observed in adult GATA6 mutant mice. In addition to the functional defect of islet cells, it may be related to age-related metabolic stress increase and associated weight gain [30]. Whether the prerequisite daughter will progress to diabetes requires further follow-up, and the early initiation of good control of insulin and metabolic indicators may be beneficial to delay the progression of the disease.

This paper has some limitations: our cases are reported in probands presenting with pancreatic abnormalities, causing bias due to selective inclusion. Then, a part of GATA6 mutation-positive individuals were derived from cohort studies where patients lack of detailed clinical evaluation about other associated features. As it is impossible to detect how extensive the clinical phenotype was in each patient, when there was no mention of pancreatic or other extrapancreatic features, we scored these data as unavailable. For family members we assumed that, if there was a pancreatic or other extrapancreatic feature described in the proband, this was also detected in carriers from the same family. If an abnormality was not mentioned in these family members, we scored this data as normal.

We hope that this study will enable treating physicians to increase the understanding of monogenic diabetes with GATA6 mutations. Subsequently, these patients should be provided for appropriate diagnostic testing and genetic counseling. Conversely, patients in whom a GATA6 pathogenic mutation has been identified should undergo a comprehensive clinical assessment and follow-up, in order to study the full disease spectrum in these individuals. Moreover, genetic testing and clinical analysis for members in family with inherent GATA6 pathogenic mutation will hopefully contribute to a better estimation of the mutational yield and to clear genotype–phenotype correlations.

### Supplementary Information

Below is the link to the electronic supplementary material.

Supplementary file1 (PDF 59 KB) Table S1 Overview of all described GATA6 mutations with pancreatic dysfunction and/or developmental defect

Supplementary file1 (DOCX 14 KB) Table S2 Differences between de novo vs. inherited mutation in the terms of pancreatic dysfunction and/or developmental defect; Table S3 Differences between missense and LoF mutation in the terms of pancreatic dysfunction and/or developmental defect
