# Supplementary material for: Monogenic Diabetes with GATA6 Mutations: Characterization of a Novel Family and a Comprehensive Analysis of the GATA6 Clinical and Genetics Traits
Source: Mol Biotechnol. 2023 May 18;66(3):467–74. doi: 10.1007/s12033-023-00761-8 (PMC10881634; doi:10.1007/s12033-023-00761-8)
Supplement: Supplementary file 1 — Supplementary file1 (PDF 59 KB) Table S1 Overview of all described GATA6 mutations with pancreatic dysfunction and/or developmental defect [file 12033_2023_761_MOESM1_ESM.pdf]

GATA6 Mutation	Protein change	Mutation type	N carriers (N probands)	Pancreatic features	Cardiac features	Other features	Inheritance	References
c.606_609dup	p.(His204fs)	Frameshift	1(1)	PD<br>NDM<br>EPI	PTA<br>PDA	Gallbladder agenesis<br>Anomalous hepatic<br>Blood flow and patent ductus venous<br>Hydronephrosis/-ureter	ND	(Stanescu, Hughes, Patel, & De León, 2015)
c.635_660del	p.(Pro212fs)	Frameshift	4(1)	PD<br>NDM<br>EPI	PDA<br>PS<br>TOF	Gallbladder agenesis<br>CDH<br>Intestinal malrotation	Inherited(maternal)	(Yau et al. 2017)
c.701del	p.(Pro234fs)	Frameshift	1(1)	PD<br>NDM<br>EPI	ASD<br>VSD	None	de novo	(Allen et al.2011)
c.705C > G	P.(Tyr235Ter)	Nonsense	1(1)	PD<br>CDM	ASD<br>VSD<br>PDA	Scoliosis	de novo	(Sanchez-Lechuga et al. 2020)
c744del	p.(Pro249fs)	Frameshift	1(1)	PD<br>NDM<br>EPI	PTA<br>HPA<br>ASD<br>VSD	Gallbladder agenesis<br>PLE<br>Developmental delay<br>Inguinal hernia<br>Congenital hip dysplasia	de novo	(McMillan, Girgis, & Sellers, 2016)
c.754_905del	p.(Ala252fs)	Frameshift	1(1)	PD<br>NDM<br>EPI	TOF	None	de novo	(Gong et al., 2013)
c.877_880delinsTAC	p.(Val293fs)	Frameshift	1(1)	PD<br>NDM<br>EPI	TOF	Biliary atresia<br>Microcephaly<br>Learning difficulties<br>Inguinal hernia	ND	(Allen et al., 2011)
c.899_902dup	p.(Ala302fs)	Frameshift	2(1)	PD<br>NDM<br>EPI	DORV<br>VSD<br>PS	Gallbladder agenesis<br>Paucity of intrahepatic bile ducts<br>Secondary hypothyroidism Bilateral posterior embryotoxon	Inherited(maternal)	(Ferreira, Devadason, Denvir, Seale,& Gupte, 2017)
c.951_954dup	p.(Leu319fs)	Frameshift	1(1)	PD<br>NDM<br>EPI	PTA<br>ASD<br>VSD<br>TI<br>TGA	Gallbladder agenesis<br>Partial biliary atresia<br>Symmestric cortical atrophy	ND	(Gong et al., 2013)
c.964_970del	p.(Tyr323fs)	Frameshift	1(1)	PD<br>NDM<br>EPI	MS<br>PDA	Gallbladder agenesis<br>Biliary atresia	de novo	(Chao et al., 2015)
c.968dup	p.(Tyr323*fs)	Frameshift	1(1)	PD<br>NDM<br>EPI	VSD<br>ASD<br>PS	Abnormal mesenteric veins	de novo	(Eifes et al., 2013)
c.969C>A	p.(Tyr323*)	Nonsense	2(1)	PD<br>NDM<br>ADM<br>EPI	ASD<br>PDA	CDH	Inherited(paternal)	(De Franco et al., 2013)
c.1036_1042del	p.(Thr346fs)	Frameshift	2(1)	PD<br>NDM<br>ADM<br>EPI	TOF	Hypothyroidism<br>Learning difficulties	Inherited(paternal)	(De Franco et al., 2013)
c.1108_1121dup	p.(Gly375fs)	Frameshift	1(1)	PD<br>NDM<br>EPI	TOF	None	de novo	(Allen et al., 2011)
c.1136-2A>G		Splicing	2(1)	PD<br>NDM<br>CDM<br>EPI	PDA	Hepatic dysfunction	Inherited(paternal)	(De Franco et al., 2013)
c.1242C>A	p.(Cys414*)	Nonsense	2(2)	PD<br>NDM<br>ADM<br>EPI	ASD<br>ASD<br>PS<br>PDA<br>TGA<br>Tricuspid atresia	Renal dysfunction (persistent proteinuria)	de novo*2	(Tuhan et al., 2015 & Michelle L. Miles et al.,2020)
c.1291C > T	p.(Gln431*)	Nonsense	2(1)	PD<br>NDM<br>ADM	ASD<br>VSD<br>PDA<br>PS	Gallbladder agenesis<br>CDH<br>Scoliosis	Inherited(maternal)	(Odile Gaisl et al.2019)
c.1303-10C>G		Splicing	1(1)	PD<br>NDM<br>EPI	IAA	Gallbladder agenesis	de novo	(Allen et al., 2011)

c.1303-1G>T		Splicing	2(1)	NDM<br>EPI	ASD<br>PS	Developmental delay<br>Hypothyroidism	Inherited(maternal)	(De Franco et al., 2013)
c.1330T>C	p.Cys444Arg	Missense	3(1)	PD<br>NDM<br>ADM<br>EPI	DORV<br>PS<br>PDA<br>VSD<br>ASD<br>PTA	Hypoganadism<br>Growth hormone deficiency<br>Umbilical hernia<br>Bifid left pelvicalyceal system<br>Right hydrocele<br>Right epididymal cyst<br>Proteinuria<br>Dysmorphic features<br>Mild-moderate Neurocognitive impairment<br>Hypospadias	Inherited(paternal)	(Yang Timothy Du, 2020)
c.1339T>C	p.(Cys447Arg)	Missense	1(1)	NDM	None	Hypothyroidism	ND	(De Franco et al., 2013)
c.1354A>G	p.(Thr452Ala)	Missense	1(1)	PD<br>NDM<br>EPI	ASD	Developmental delay<br>Intestinal perforation	de novo	(Allen et al., 2011)
c.1366C>T	p.(Arg456Cys)	Missense	4(4)	PD<br>NDM<br>CDM<br>EPI	PTA<br>VSD<br>TOF<br>IAA	Umbilical hernia<br>Developmental delay<br>Seizures<br>Hypothyroidism<br>Absent gall bladder<br>Thrombocytopenia<br>Neonatal stroke<br>Adrenal insufficiency	de novo*4	(Nikhil Raghuram et al.202,Allen et al.2011,Sanyoura et al.2018)
c.1367G>A	p.(Arg456His	Missense	2(2)	PD<br>NDM<br>ADM<br>EPI	PDA<br>VSD<br>HLPA	Developmental delay<br>Gallbladder agenesis<br>Renal duplex collecting system	de novo*2	(Allenby al,201,Doris Škoric-Milosavljevet al.2019)
c.1396A>G	p.(Asn466Asp)	Missense	1(1)	PD<br>NDM<br>EPI	PDA	Gallbladder agenesis<br>Intestinal malrotation<br>Microcolon<br>Developmental delay<br>Epilepsy<br>Transient hypothyroidism	ND	(Allen et al, 2011)
c.1397A>G	p.(Asn466Ser)	Missense	1(1)	PD<br>NDM	ASD<br>PS<br>PDA	Transient idiopathic neonatal cholestasis<br>Hypoglycemia episodes<br>Low growth and cortisol level	de novo	(Catli et al., 2013)
c.1399G>A	p.(Ala467Thr)	Missense	1(1)	PD<br>NDM<br>EPI	ASD<br>PS	Pituitary agenesis<br>Learning difficulties<br>Seizures	ND	(Allen et al., 2011)
c.1406G>A	p.(Gly469Glu)	Missense	2(1)	PD<br>NDM<br>ADM<br>EPI	None	Hepatomegaly<br>Developmental delay<br>Hemiplegia<br>Hypothyroidism	Inherited(paternal)	(De Franco et al., 2013)
c.1417A>C	p.(Lys473Gln)	Missense	1(1)	PD<br>NDM<br>EPI	ASD	Gallbladder agenesis	de novo	(Allen et al., 2011)
c.1428+1G>T		Splicing	1(1)	PD<br>NDM	ASD<br>PDA	Developmental delay	de novo	(Chao et al., 2015)

				EPI		Anaemia		
c.1429-41_1441del		Splicing	1(1)	PD<br>NDM<br>EPI	PDA	None	de novo	(De Franco et al., 2013)
c.1429-8T>G		Splicing	1(1)	PD<br>NDM<br>EPI	Dextrocardia<br>APW<br>AVSD	Early cognitive and motor delay<br>CDH	de novo	(De Franco et al., 2013)
c.1435A>G	p.(Arg479Gly)	Missense	1(1)	NDM<br>ADM<br>EPI	TGA<br>VSD<br>PDA	Cryptorchidism<br>PLE	de novo	(De Franco et al., 2013,Doris Škori_x0019_c-Milosavljev et al.2019)
c.1448_1455del	p.(Met483fs)	Frameshift	1(1)	PD<br>NDM<br>EPI	ASD<br>VSD<br>PS<br>PDA<br>HRV<br>HTV	Gallbladder agensis	de novo	(Allen et al., 2011)
c.1477C>T	p.(Arg493*)	Nonsense	1(1)	PD<br>NDM<br>EPI	VSD<br>PDA	CDH	de novo	(Suzuki et al., 2014)
c.1498_1501del	p.(Lys500fs)	Frameshift	1(1)	PD<br>NDM<br>EPI	DOLV<br>VSD<br>HPA<br>PS<br>PFO<br>PDA	None	de novo	(Allen et al., 2011)
c.1504_1505del	p.(Lys502fs),<br>p.(K562Dfs)	Frameshift	4(2)	PD<br>NDM<br>CDM<br>EPI	VSD<br>TOF<br>PDA	Gallbladder agenesis<br>Bicornuate uterus<br>Neonatal pancytopenia<br>Hypercativity	1*Inherited(maternal)<br>1*ND	(Bonnefond et al., 2012.Yorifuji et al., 2012)
c.1516+1G>C		Splicing	1(1)	PD<br>NDM<br>EPI	ASD<br>PFO	transient hypothyroidism	de novo	(Allen et al., 2011)
c.1516+4A>G		Splicing	1(1)	PD<br>NDM<br>EPI	None	CDH	de novo	(Allen et al., 2011)

Table S. Overview of all described GATA6 mutations with pancreatic dysfunction and/or developmental defect.

List of abbreviations (alphabetically ordered):

ADM: Adult-onset Diabetes Mellitus
ASD: Atrial Septal Defect
APW: Aortopulmonary Window
AVSD: Atrioventricular Septal Defect
CDH: Congenital Diaphragmatic Hernia
CDM: Children-onset Diabetes Mellitus
DOLV: Double Outlet Left Ventricle
DORV: Double-outlet right ventricle
EPI: Exocrine Pancreatic Insufficiency
HLPA: Hypoplastic Left Pulmonary Artery
HPA: Hypoplastic Pulmonary Artery
HRV: Hypoplastic Right Ventricle
HTV: Hypoplastic Tricuspid Valve
IAA: Interrupted Aortic Arch
MS: Mitral valve Stenosis
NDM: Neonatal Diabetes Mellitus
PD: Pancreatic Dysplasia
PDA: Patent Ductus Arteriosus
PFO: Patent Forum Ovale
PLE: Protein Losing Enteropathy
PS: Pulmonary Stenosis
PTA: Persistent truncus arteriosus
TGA: Transposition of the Great Arteries
TI: Tricuspid valve Insufficiency
TOF: Tetralogy Of Fallot
VSD: Ventricular Septal Defect
