# Supplementary material for: Monogenic Diabetes with GATA6 Mutations: Characterization of a Novel Family and a Comprehensive Analysis of the GATA6 Clinical and Genetics Traits
Source: Mol Biotechnol. 2023 May 18;66(3):467–74. doi: 10.1007/s12033-023-00761-8 (PMC10881634; doi:10.1007/s12033-023-00761-8)
Supplement: Supplementary file 2 — Supplementary file1 (DOCX 14 KB) Table S2 Differences between de novo vs. inherited mutation in the terms of pancreatic dysfunction and/or developmental defect; Table S3 Differences between missense and LoF mutation in the terms of pancreatic dysfunction and/or developmental defect [file 12033_2023_761_MOESM2_ESM.docx]

|  | De novo（N=28）n(%) | Inherited（N=24）n(%) | P value |
| --- | --- | --- | --- |
| NDM | 24（85.7） | 10（41.7） | 0.00** |
| CDM | 5（17.9） | 5（20.8） | 0.53 |
| ADM | 0（0） | 4（16.7） | 0.04 |
| PD | 27（96.4） | 13（54.2） | 0.00** |
| EPI | 22（78.6） | 10（41.7） | 0.01 |

Table S2 Differences between de novo vs. inherited mutation in the terms of pancreatic dysfunction and/or developmental defect.

|  | Missense（N=18）n(%) | LOF（N=41）  n(%) | P value |
| --- | --- | --- | --- |
| NDM | 13（72.2） | 27（65.9） | 0.54 |
| CDM | 3（16.7） | 8（19.5） | 0.74 |
| ADM | 2（11.1） | 2（48.8） | 0.30 |
| PD | 15（83.3） | 31（75.6%） | 0.51 |
| EPI | 11（61.1） | 27（65.9） | 0.56 |

Table S3 Differences between missense vs. LoF mutation in the terms of pancreatic dysfunction and/or developmental defect.

**Statistical comparisons were performed using Fisher's exact test, and Bonferroni corrected p values were used for determination of statistical significance (p =0 .01).

List of abbreviations (alphabetically ordered):

ADM: Adult-onset Diabetes Mellitus

CDM: Children-onset Diabetes Mellitus

EPI: Exocrine Pancreatic Insufficiency

NDM: Neonatal Diabetes Mellitus

PD: Pancreatic Dysplasia
